# Application of Raman spectroscopy and Machine Learning algorithms for fruit distillates discrimination

**DOI:** 10.1038/s41598-020-78159-8

**Published:** 2020-12-03

**Authors:** Camelia Berghian-Grosan, Dana Alina Magdas

**Affiliations:** National Institute for Research and Development of Isotopic and Molecular Technologies, 67-103 Donat Str., 400293 Cluj-Napoca, Romania

**Keywords:** Metabolomics, Applied physics

## Abstract

Through this pilot study, the association between Raman spectroscopy and Machine Learning algorithms were used for the first time with the purpose of distillates differentiation with respect to trademark, geographical and botanical origin. Two spectral Raman ranges (region I—200–600 cm^−1^ and region II—1200–1400 cm^−1^) appeared to have the higher discrimination potential for the investigated distillates. The proposed approach proved to be a very effective one for trademark fingerprint differentiation, a model accuracy of 95.5% being obtained (only one sample was misclassified). A comparable model accuracy (90.9%) was achieved for the geographical discrimination of the fruit spirits which can be considered as a very good one taking into account that this classification was made inside Transylvania region, among neighbouring areas. Because the trademark fingerprint is the prevailing one, the successfully distillate type differentiation, with respect to the fruit variety, was possible to be made only inside of each producing entity.

## Introduction

Fruit distillates represent an alcoholic beverage from Central and East European countries which traditional production implies the distillation of various fermented fruits (especially plums, but also apples, pears, apricots, etc.). Depending of the regions from Romania, these beverages are known as “*ţuică*”, “*pălincă*” or “*horincă*”. Considering the ethanolic concentration, their alcoholic strength varies from 24 to 86% v/v for “*“ţuică”*” and from 40 to 70% v/v for “*pălincă*”^1^, or they can be differentiated as function of the fruits variety: “*ţuică*” and “*horincă*” for spirits obtained from plums and “*pălincă*” for that achieved from other fruits (apples, apricots, pears, cherries, etc.)^2^.

Regardless of their commercial name, the composition of traditional Romanian distillates is a very complex one^2^, being influenced by the botanical origin alongside with the provenience region of fruits. To this, the preparation technologies and ageing in different wood barrels also affect the chemical constituents from fruit distillates, all of them influencing their quality^3^. In Transylvania, the knowledge related to the distillates production process represents a legacy from father to sons. Not only that this tradition was proudly kept during the history, but it also has been enriched and perfected during the time. Nowadays, the continuous improvement of the distillation knowledge, in order to obtain premium products, is intertwined with keeping the traditions, passion for this profession and art.

In order to encourage and sustain the producing of high-quality fruit spirits and to detect fraudulently attempts, like false declaration of product provenience, new approaches should be established. The development of fast, reliable and economical effective analytical methods able to differentiate among distinct food and beverages categories became a priority for research and control entities during the years. This interest was doubled by the producers’ perspective which are willing to keep the control on the quality and fingerprint of the goods they are producing. Therefore, the development of control methodologies, easy to be applied from the measurement’s skills perspective, became essential. In this context, vibrational spectroscopy techniques (IR and Raman) appear to be the ideal candidate, especially because of the development of new portable devices easily to be operated, and also due to the fact that through these techniques the measurements are performed directly on the sample. The vibrational spectroscopy was generally used for quantitative determination of ethanol and/or methanol from the fruit distillates^1,4–7^. As compared with IR methods, Raman spectroscopy is suitable for the analysis of high-water content food products because of its relatively weak water bending mode in the fingerprint region^8^.

Taking into account that spectroscopic methods generate large data sets, an advanced data processing is mandatory to extract meaningful information. An effective approach is given by the association between Raman spectroscopy and Machine Learning algorithms in order to discriminate between different constituents of complex substances^9^. Thus, this methods association was successfully applied in different fields like: food analysis^10,11^, bacteria identification^12^ or even diagnostic applications^13^.

In this context, the aim of this study was to test the potential of the application of Raman fingerprint, in conjunction with Machine Learning algorithms, for fruit distillates classifications. The three differentiation criteria which were followed alongside this study were: (i) the fruit variety which was used as row material; (ii) geographical origin; (iii) trademark fingerprint.

## Materials and methods

### Sample description

All fruit distillates were provided by eight Romanian producers (30 samples). Two large producers, further denoted as processing companies (PC), supplied different varieties of fruit distillates as follows: PC 1–5 samples (apples, apricots, pears, plums, quince); PC 2–6 samples (apples, pears, plums, quince). Three small producers, designated as manufactures (MF), supplied the following samples: MF 1–5 samples (apricots, cherries, pears, plums, sour-cherries); MF 2–4 samples (apples, apricots, plums) and MF 3–7 samples (apples, grapes, plums). These alcoholic beverages were originated from four Transylvanian regions (Bistrita Nasaud—BN; Covasna—CV; Salaj—SJ; Satu Mare—SM). To these, a control sample set formed by three samples (2 plums and 1 pears distillates) from three small producers (manufactures) of Salaj (SJ) region was added to test the prediction capability of the model built for geographical origin recognition. Alcoholic strength of the fruit distillates was determined by GC-FID (PerkinElmer 990).

### Raman measurements and data processing

A JASCO NRS-3300 equipped with a CCD detector (− 69 °C) was employed for the Raman measurements. A diode laser system emitting at 785 nm wavelength, 600 lines/mm grating and an UMPLFL Olympus objective of 20× were used for recording the Raman spectra. The calibration was performed using the sharp peak of Si from 521 cm^−1^. For the experiments, 4 mL of fruit distillates were placed in a glass vessel; the spectrum was recorded using 100 s as exposure time and 3 accumulations.

The JASCO Spectra Manager (JASCO, Easton, USA) tools were used for spectra analysis and selection of the frequency range (120–1700 cm^−1^) before any processing of the Raman data. Then, for each sample, the average spectrum (obtained using the statistics on rows, mean process for the spectra registered in two points) was subjected to the baseline subtraction and the [0,1] normalization. These processes were realized in OriginPro 2017 (OriginLab, Northampton, USA) and allowed a fair comparison of the samples, especially of those manifesting the fluorescence phenomenon. These Raman data were further employed both for general Raman and Machine Learning studies.

### Machine Learning investigations

Machine Learning investigations were performed using the Classification learner app implemented in MATLAB R2018b (MathWorks, Natick, Massachusetts, USA) and the pre-treated Raman spectra of fruit distillates in the range 120–1700 cm^−1^. Considering the botanical, producers or geographical differentiation challenges, different training and testing groups have been adopted, all these being clearly indicated in each corresponding section. In order to study the use of Raman spectroscopy and Machine Learning algorithms for several fruit distillates discrimination, the five predictive modelling approaches were used: the decision trees^14^, the discriminant analysis^15^, the support vector machines (SVM)^16^, the nearest neighbour classifiers (KNN)^17^, ensemble classifiers^18^.

### Ethical approval

This article does not contain any studies with human participants or animals performed by any of authors.

## Results

**Figure 1:**
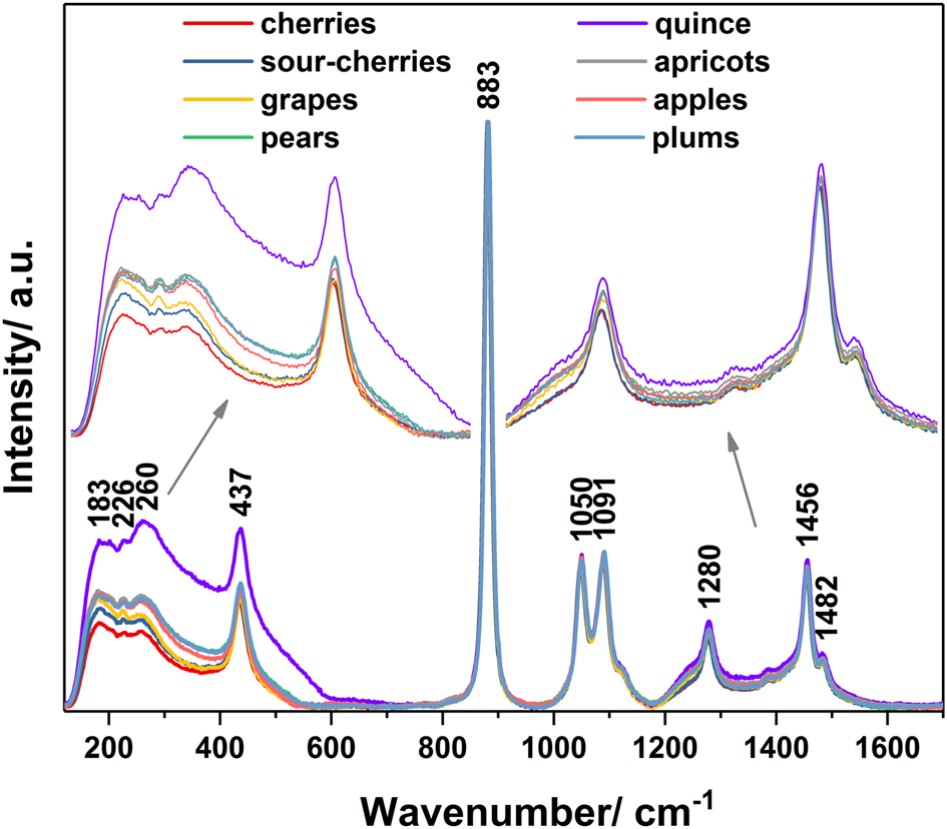
Raman spectra of distillates obtained from different varieties of fruit.

Figure 1 contains the Raman spectra of the eight fruit distillates varieties. These fruit spirits contain between 40 and 80 percent alcohol by volume and were obtained based on different fruit (apple, apricot, cherry, grape, pear, plum, quince, sour-cherry). The main Raman peaks, illustrated in Fig. 1 and assigned in Table 1, can be associated with the ethanol vibrations^5,19–21^. Some of these bands, namely 883, 1050 and 1456 cm^−1^, are generally used as single or multiple-band normalization method for quantification of ethanol in alcoholic beverages^22,23^.

**Table 1:** Main Raman peaks and their assignments.

Main Raman peaks/cm^−1^	Peaks’ assignments^5,19–21^
183	Lattice mode
226	**–**
260	**–**
437	C**–**C**–**O in-plane bending
883	C**–**C stretching
1050	C**–**O stretching
1091	CH_3_ rocking
1280	CH_2_ torsion and rotational vibrations
1456	CH3 bending
1482	CH3 bending

A brief analysis of Fig. 1 indicates the existence of two ranges (region I—200–600 cm^−1^ and region II—1200–1400 cm^−1^) with small differences among spectra that can be the consequence of different influences like: producer technologies, geographical origin or fruit varieties. Thus, the presence of metals, Cu, Zn, Fe, Al, etc. from various sources (i.e. raw materials, process type, storage conditions)^24^ and the volatile compounds, like esters^2^ could affect the Raman profile of the alcoholic beverages, having some characteristic bands (Metal–O, Metal–C and C–O–C respectively) in these regions^25^.

**Figure 2:**
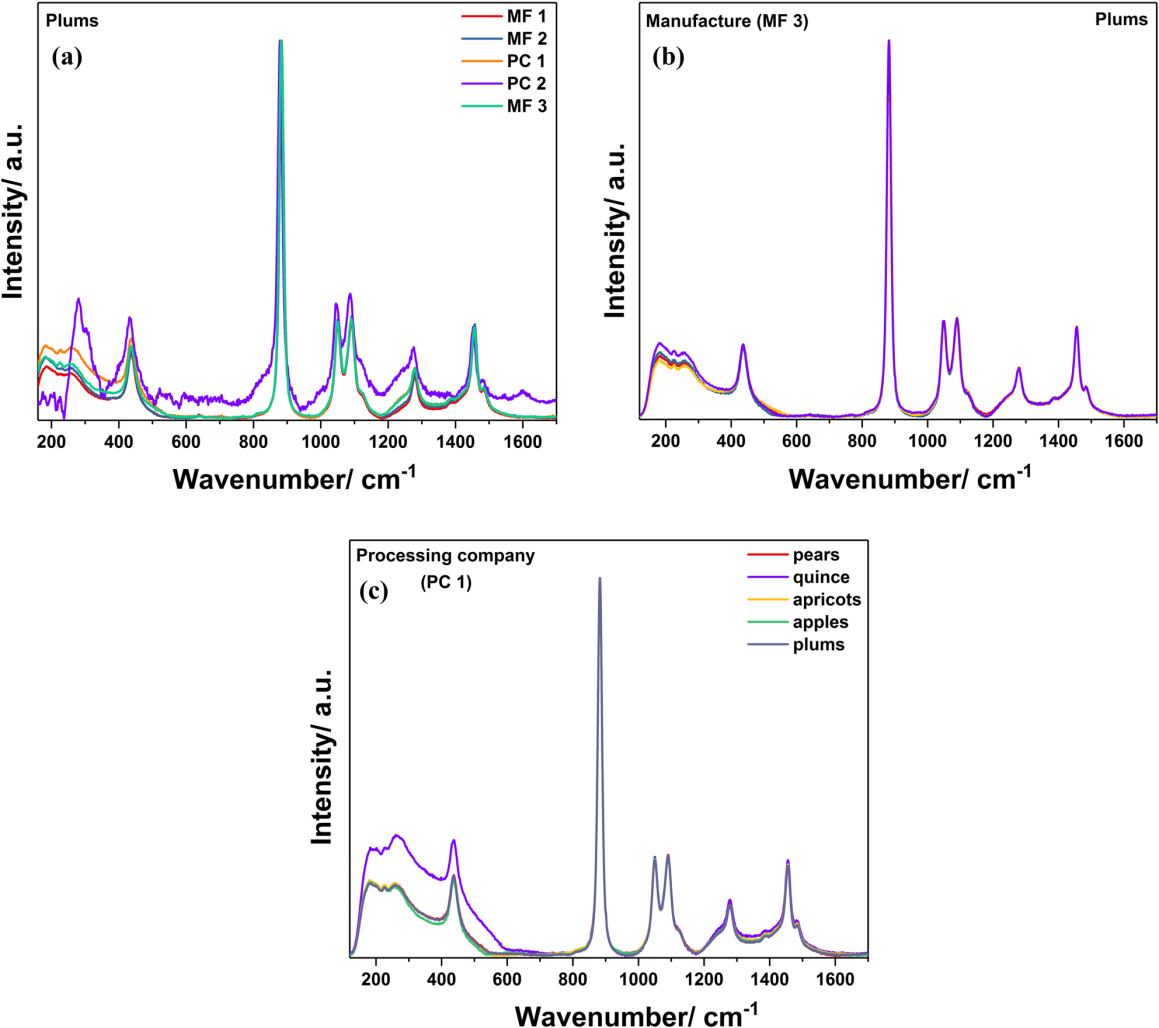
Raman spectra of plums distillates considering the influence of the production process (**a**,**b**) and impact of fruit varieties onto the Raman spectra of PC 1 distillates’ samples (**c**).

The investigation of the Raman spectra of five plums distillates purchased from five different spirits producers shows differences in the same two regions, 200–600 and 1200–1400 cm^−1^ (Fig. 2a). In this figure, the different Raman pattern of the plums spirit from PC 2 is mainly explained through the great fluorescence of the sample, which could be primarily the result of the storage conditions used by this producer^26^. Going further and analysing the spectra of plums distillates obtained from one manufacture, i.e. MF 3 (Fig. 2b), very slight differences in the region of 200–600 cm^−1^ can be observed. Moreover, the obtained data for five fruit varieties spirits from PC 1 (Fig. 2c) highlighted small changes in the spectral region 1200–1400 cm^−1^, while the spectrum obtained for quince spirit is the result of fluorescence influence due to the specific, light yellow colour of this alcoholic beverage.

Because of these very subtle changes which appear among the investigated samples, which are sometimes very difficult to be estimated only by eyes, the use of an advanced data processing tool was necessary to be employed. For this purpose, Machine Learning algorithms were used for the differentiation among distinct classes like: botanical and geographical origin as well as for trademark identification.

## Discussions

### Prevailing influences on the Raman fingerprint of distillates: fruit variety vs. final product characteristics

The first performed differentiation on the investigated spirits aimed to discriminate the fruit variety from which each distillate was obtained. For this classification, a number of 27 fruit distillates (apricots, cherries, pears, plums, quince, sour-cherries) produced in two processing companies (PC) and three manufactures (MF) were involved. The distillates which were purchases from each producer were the following: PC 1 (apples, apricots, pears, plums, quince); PC 2 (apples, pears, plums, quince); MF 1 (apricots, cherries, pears, plums, sour-cherries); MF 2 (apples, apricots, plums) and MF 3 (apples, grapes, plums). Before the investigation, a training set containing the Raman spectra of 22 fruit distillates’ samples, assuring the representativeness of each producers, was created for further data processing. Based on these experimental data, the ML algorithms extracted the essential information in order to build the classification model. Other 5 spectra of randomly selected fruit distillates’ samples were employed for the testing set generation. This group was created to verify the prediction of the model obtained on the training set and has the role of external sample quality control^9^. Thus, considering the fruit variety criterion, the best obtained accuracy was of only 27.3% being achieved based on the model Ensemble (boosted trees), suggesting that no differentiation can be made in this case. In these conditions, the model verification, using the testing set, was not relevant anymore, therefore it was not performed.

It is well known that the technological process as well as the storage conditions highly impact the fruit distillates overall composition and their quality. Thus, in order to verify if a Raman fingerprint of the final product, can be link with a certain producer, a new classification of the fruit distillates as function of PC/MF, was performed.

**Figure 3:**
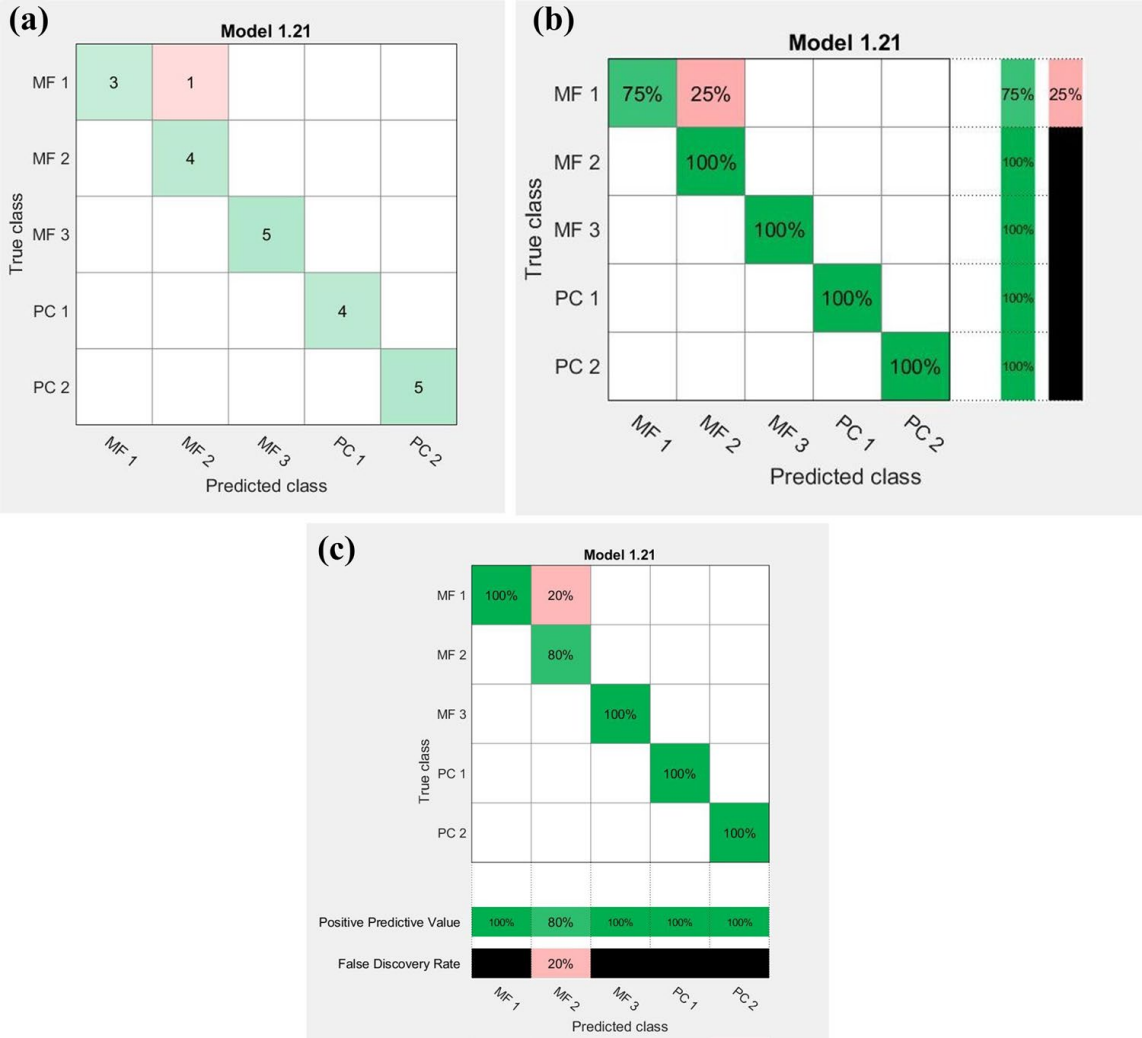
Confusion matrix obtained for the fruit distillates considering the producers’ influences; classification presented as number of observations (**a**), true positive vs. false negative rates (**b**) or positive predicted values vs. false discovery rate (**c**).

For this purpose, a new prediction model was obtained by applying all the classification learner algorithms from Matlab 2018b onto the training and testing groups previously created, for the fruit variety study. As can be observed in Fig. 3, independently of the distillate type (fruit variety), a high capacity for separation among producers, especially processing companies (PC) and two manufactures (MF), was obtained. This fact clearly demonstrates that the main influence on the Raman fingerprint of the distillates is given by the spirits processing and storage conditions rather than the raw material employed in the process. As can be seen from Fig. 3, only one sample from MF 1 was wrong attributed to MF 2. A possible explanation in this regard could be related to the similarities in the production processes between the two manufactures taking into account that both of them belongs to the same family-owned business, following the same traditional manufacturing steps.

Due to the high accuracy (95.5%) of the classification model, Ensemble (subspace KNN), its evaluation was realized on a testing group, containing 5 Raman spectra of randomly selected samples, inside of four producers. The testing set was built as follows: one sample from PC 1 and PC 2, one sample from MF 1 and two samples from MF 3; on account of the few samples acquired from MF 2, this manufacture was not included in the testing set. The results show a good capacity of the model to correctly predict the appurtenance of the tested samples (each sample from the testing set has been assigned to the right PC or MF).

The main question which arose here was if the classification among fruit distillates producers was made based on its specific fingerprint or was related to the ethanol concentrations. This because, as can be seen from Fig. 1, the main signals which appear in Raman spectra are those given by the ethanol (Table 1). Therefore, in order to better understand which is the connection between the distillates’ producers and Raman fingerprint and if this relationship is not influenced by the ethanol concentrations, a classification of distillates as function of their alcoholic strength was performed.

**Figure 4:**
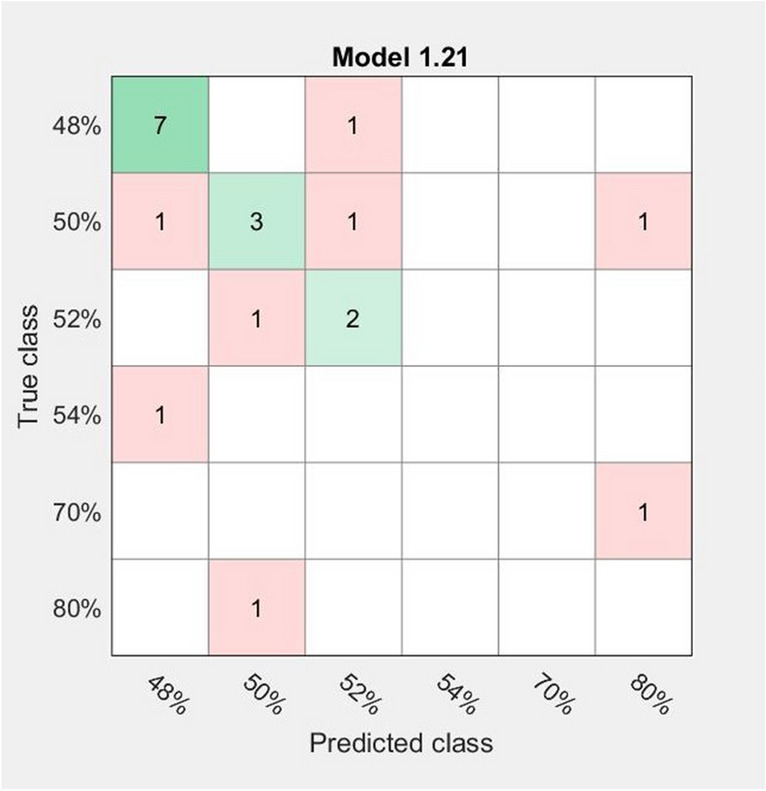
Confusion matrix obtained for the fruit distillates investigation considering the influence of the ethanol concentration.

For this purpose, the classification was carried out on a training set containing 20 samples, having the following alcoholic concentrations: 80% (one sample), 70% (one sample), 54% (one sample), 52% (three samples), 50% (six samples), 48% (eight samples), while the testing set implied 7 samples of 52% (one sample), 50% (one sample) and 48% (three samples). The obtained results are presented in Fig. 4 and indicate a small differentiation between the 6 classes of the investigated alcoholic concentrations. The best response (accuracy 60%) was obtained for an Ensemble model (subspace KNN), and because of the low achieved classification percentage the verification of the model with the test dataset was not further made.

For this differentiation, a poor correlation was obtained suggesting that a producer Raman fingerprint exists independently of the ethanol concentrations. All these results highlight the idea that the discrimination among the investigated distillates is not linked to the major Raman peaks, but rather to the minor components containing in these alcoholic beverages.

### Distillates’ classification considering their botanical origin inside of each producer

To test if a discrimination as function of fruit variety can be achieved, after exclusion of the trademark effect, a new classification series was performed inside of each fruit distillates producer.

**Figure 5:**
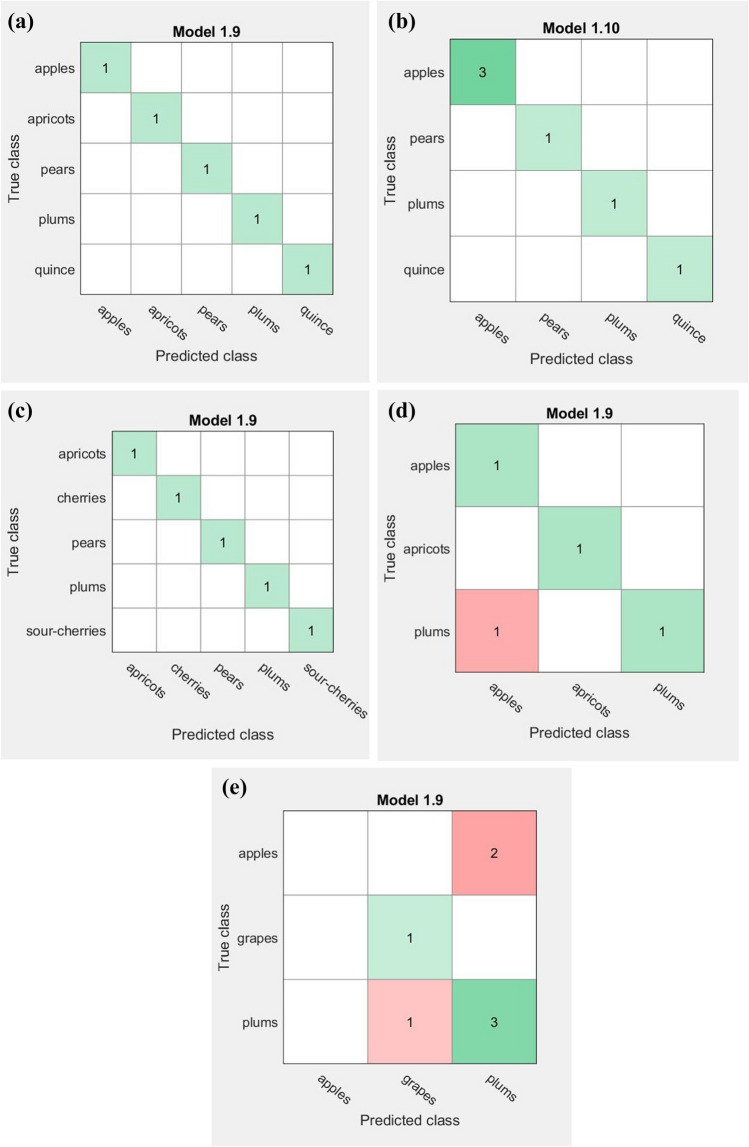
Confusion matrix obtained for the fruits distillates’ classification considering their botanical origin inside of each producer; classification presented as the number of observations for PC 1 (**a**), PC 2 (**b**), MF 1 (**c**), MF 2 (**d**) and MF 3 (**e**) producers.

In this study, for each producer a training set containing all the samples owned from that producer was created. Thus, five training sets that include a total of 27 samples were used by the Machine Learning algorithms to build the appropriate models (Fig. 5a–e). Due to the low number of the same fruit variety inside each producer, the testing step was not possible to be performed for these classifications. Based on the obtained results, good discrimination of fruit type inside the processing companies and a relatively acceptable differentiation of the fruit varieties inside the manufactures, we consider that the prediction models could be successfully used for this type of analysis. The high accuracy (100%) of the models (fine Gaussian SVM and medium Gaussian SVM, respectively) achieved for the processing companies (PC) might be due to a more rigorous and constant technological process as well as to similar storage conditions for all distillates. The same method (fine Gaussian SVM) yielded a high accuracy (100%) for MF 1 and 75% or 57.1% for MF 2 and MF 3 respectively. These results could suggest that for an accurate identification of fruit fingerprint inside the producer distillates, each producer should follow similar technological and storage conditions for its fruit spirits.

### Distillates’ classification considering their geographical origin

For the geographical differentiation, samples from four Transylvanian regions were used (Bistrita Nasaud—BN; Covasna—CV; Salaj—SJ; Satu Mare—SM). From SM region, samples from one distillate processor and one manufacture were involved in the classification: PC 2 and MF 1.

**Figure 6:**
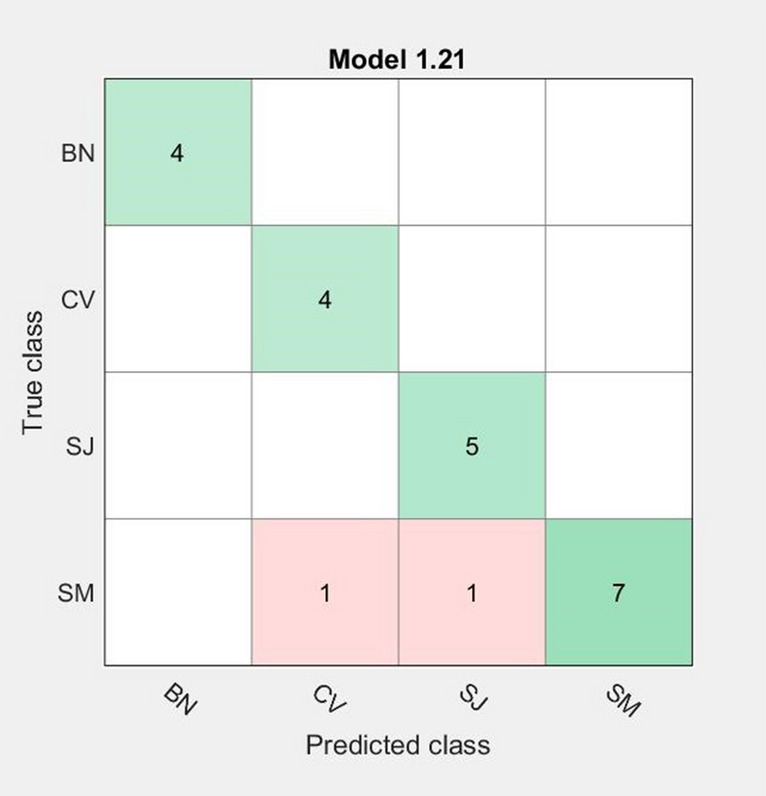
Confusion matrix obtained for the fruit distillates’ classification considering their geographical origin.

Within this analysis, the training set was formed by the 22 Raman spectra of the fruit distillates’ samples generally used for the fruit variety and producers’ discrimination (Fig. 6). The best geographical classification of the fruit distillates was obtained with the Ensemble (subspace KNN) method—accuracy 90.9% (two samples were misclassified). For the testing dataset, 3 more Raman spectra were added to that of the 5 distillates’ samples contained in the previously mentioned classifications in order to enlarge the geographical groups, even if the new spirits could not be correlated with the investigated producers. Thus, a total of 30 fruit distillates were employed for the geographical investigations. The testing set consisted of the following samples: 2 from SM, 1 from BN and 5 from SJ. The obtained results showed a good correlation of the predicted regions with the true investigated ones. Only one sample from SJ country was misclassified and assigned to BN region, while the other 7 samples were correctly predicted even if the producers of three of them were new. Considering that this classification was made inside Transylvania region, among neighbouring areas, these results are very promising.

## Conclusions

This pilot study revelled the existence of a specific distillate producer fingerprint which can be pointed out through the association between Raman spectroscopy and Machine Learning algorithms. The trademark fingerprint dominates the varietal one, proving the high influence which is manifested through the entirely production and storage processes on the Raman spectra of the distillates. Anyway, the fruit variety classification of distillates was possible to be successfully performed inside of each producer, only after the technological influences were eliminated.

The classification model built for geographical recognition proved to be effective for the correct attribution of seven samples from the eight investigated ones, even if some of these samples were purchased from other distillates producers.

Through this work it was demonstrated the potential offered by association between Raman spectroscopy and Machine Learning algorithms for a rapid and unexpansive way to verify the fruit distillates trademarks.
